# 656. Single Set Blood Cultures: A Set Short or One Set Too Many?

**DOI:** 10.1093/ofid/ofaf695.211

**Published:** 2026-01-11

**Authors:** Patrick R Ching, Barry Rittmann, Jett Choquette, Alexandra L Bryson, Christopher Doern, Michelle Doll

**Affiliations:** Virginia Commonwealth University School of Medicine, Richmond, Virginia; Virginia Commonwealth University Health System, Richmond, Virginia; Virginia Commonwealth University Medical Center, Richmond, Virginia; Virginia Commonwealth University Health System, Richmond, Virginia; Virginia Commonwealth University Health System, Richmond, Virginia; Virginia Commonwealth University Health System, Richmond, Virginia

## Abstract

**Background:**

The 2023 blood culture (BC) shortage pushed hospitals to rapidly implement conservation measures. During the shortage, our facility encouraged single set BC (SSBC) for patients at lower risk based on an algorithm embedded in the order. Despite the end of the shortage and algorithm removal, ongoing use of SSBC was observed. This study evaluated for detectable patient harm in the form of delayed diagnosis of infection after a negative SSBC.

**Methods:**

All ED or inpatient negative SSBCs ordered on adults from Mid-June-September in 2023 and 2024 were included. The 2023 period corresponded to the implementation of conservation measures. The 2024 period was a post-shortage comparator.

The patient chart for each SSBC was manually reviewed by 3 physicians: PC, MD, JC, for the following variables: visit diagnoses, evidence of positive BC within 30 days of index SSBC, 30 day readmissions and mortality, clinical concern for potential missed bacteremia in the opinion of the reviewer, appropriateness based on an evidence-based BC stewardship algorithm.

Summary statistics were performed; data from 2023 and 2024 SSBCs were compared using Chi-square tests. Analyses were performed using SAS 9.4.

**Results:**

There were 119 SSBCs from 2023 (shortage), and 388 from 2024 (post-shortage).

No patient had documented bacteremia within 30 days of the index negative SSBC. No patient was re-admitted with infection suggesting unrecognized bacteremia.

There were 9 patients made comfort care due to end-stage organ failures or malignancies, in which the reviewer could not rule out possibility of missed bacteremia: 2 (1.7%) during the shortage, and 7 (1.8%) post-shortage (p=0.706).

The majority of SSBCs did not meet appropriateness criteria: 81/119 (68.1%) inappropriate during the shortage, and 283/388 (72.9%) post-shortage, p=0.297.

**Conclusion:**

No overt evidence of missed bacteremia (in the form of a subsequent positive BC or readmission for a previously missed infection) was detected.

Overall, 72% (364/507) of the SSBCs reviewed were deemed inappropriate applying a blood culture stewardship algorithm.

SSBCs for low risk patients may be reasonable; risk of missed bacteremia appears to be low. However, providers ordering SSBCs should consider whether a blood culture is needed at all.

**Disclosures:**

Christopher Doern, PhD, Karius, Inc: Honoraria|Roche Diagnostics: Advisor/Consultant|Shionogi Pharmacovigilance Center Co., Ltd.: Honoraria

